# Magnetostatic reciprocity for MR magnet design

**DOI:** 10.5194/mr-2-607-2021

**Published:** 2021-08-04

**Authors:** Pedro Freire Silva, Mazin Jouda, Jan G. Korvink

**Affiliations:** Karlsruhe Institute of Technology (KIT), Institute of Microstructure Technology, 76131 Karlsruhe, Germany

## Abstract

Electromagnetic reciprocity has long been a staple in magnetic resonance (MR) radio-frequency development, offering geometrical insights and a figure of merit for various resonator designs. In a similar manner, we use magnetostatic reciprocity to compute manufacturable solutions of complex magnet geometries, by establishing a quantitative metric for the placement and subsequent orientation of discrete pieces of permanent magnetic material. Based on magnetostatic theory and non-linear finite element modelling (FEM) simulations, it is shown how assembled permanent magnet setups perform in the embodiment of a variety of designs and how magnetostatic reciprocity is leveraged in the presence of difficulties associated with self-interactions, to fulfil various design objectives, including self-assembled micro-magnets, adjustable magnetic arrays, and an unbounded magnetic field intensity in a small volume, despite realistic saturation field strengths.

## 1 Introduction

Magnetic field generation is an intrinsic requirement of magnetic resonance (MR) equipment, providing both the source of Zeeman polarisation and the precession field. Despite a sharp dependence of the MR sensitivity on field intensity, the high cost of superconducting magnets has resulted in an increase in the use and development of compact permanent-magnet systems, starting in the 1980s [Bibr bib1.bibx41] with the discovery of high-remanence and high-coercivity magnetic materials, such as neodymium-based alloys. As better detection methods, hyperpolarisation techniques, and excitation schemes lower limits of detection, new applications based on these magnet systems have been made possible by offsetting the signal-to-noise ratio (SNR) loss stemming from the reduced magnetic field intensity. Beyond a cost reduction, permanent magnets have allowed for portable and non-standard MR applications and can be divided into narrow-bore spectroscopy magnets [Bibr bib1.bibx9], large-bore imaging magnets [Bibr bib1.bibx13], and other speciality magnets providing an external homogeneous volume [Bibr bib1.bibx39], profiling gradients [Bibr bib1.bibx31], or pre-polarisation fields [Bibr bib1.bibx37].

Despite the numerous published concepts, several key bottlenecks and cost factors remain, hindering the design and construction of suitably good-quality magnet systems. Reported challenges include the complexity in using tens of source magnets [Bibr bib1.bibx13], a magnetisation strength error [Bibr bib1.bibx13] of 1 %, a magnetisation direction error [Bibr bib1.bibx13] of 2 ${}^{\circ }$ , and a magnet size error [Bibr bib1.bibx32] of 0.1 mm, as well as experimental difficulties regarding assembly–alignment errors and the large forces associated with strong permanent magnets. As target inhomogeneities in MR lie below the parts-per-million (ppm) range, a priori magnet deviations of 1 % constitute a massive obstacle in achieving high-homogeneity magnets. Current methodology requires, for example, the time-consuming analysis of each magnet in a large pool, so as to use only the most similar magnets [Bibr bib1.bibx13], the individual characterisation and ordering of an array to reduce inhomogeneity [Bibr bib1.bibx13], or the use of high-precision mechanical adjustment systems [Bibr bib1.bibx9]. All of these approaches limit the number of magnets in a system and, as a result, the final field homogeneity. These efforts are often not possible or sufficient, and thus additional passive shimming techniques are required [Bibr bib1.bibx13], in addition to active shimming.

So as to overcome these limitations, and building upon the self-assembly idea mentioned in [Bibr bib1.bibx3], the long-known principle of magnetostatic reciprocity, also known as Betti's theorem [Bibr bib1.bibx15], was considered for generalised MR permanent magnet design. Geometrical reciprocity is ever-present in MR in the form of electromagnetic reciprocity from [Bibr bib1.bibx12], which creates a correspondence between a current distribution in the MR detection coil and the signal induced in it. Similarly, one can deduce a reciprocity between two regions in space and the magnets/fields contained therein. This allows for the quantitative *evaluation* of proposed magnet designs as well as for the planning of the *assembly* of the geometry. An easier alignment and assembly allows for the use of a larger number of discrete magnets, which in turn allows for the discretisation of magnetisation distributions with better fidelity, and the use of smaller, cheaper, and safer magnets [Bibr bib1.bibx41].

The principle and its direct and indirect consequences are initially discussed in Sect. [Sec Ch1.S2.SS1], subsequently enabling the computation of a material-independent figure of merit for magnetic systems in Sect. [Sec Ch1.S2.SS2]. In a similar analytical approach, the impact of an increasing number of magnets on the field quality of a Halbach array was researched, in Sect. [Sec Ch1.S2.SS3], to showcase the benefits of being able to handle a larger number of magnets.

To demonstrate the breadth of possibilities and insights allowed when designing with the principle, three different applications were proposed. The first leverages the minimum-energy state that comes with maximum magnetic coupling, for an easier auto-assembly of a Halbach magnet, and is shown in Sect. [Sec Ch1.S5.SS1]. A second application proposes an arbitrarily chosen development goal and shows how the reciprocity principle can elucidate the design process on each step, allowing for the complex behaviour shown in Sect. [Sec Ch1.S5.SS2]. The final application maximally leverages the principle, enabling a topological optimisation method to obtain the highest possible magnetic field in a single-sided magnet, which shows an unbound magnetic field strength in Sect. [Sec Ch1.S5.SS3]. The methods used for simulation, material modelling, and result post-processing are shown before the exposition of numerical results, in Sect. [Sec Ch1.S3], for clarity, and can all be implemented and run on a standard laptop within a few minutes to hours. The paper concludes with a discussion of the results and an outlook for the technique in Sect. [Sec Ch1.S6].

## 2 Theoretical insights

### 2.1 Principle corollaries

Consider an ensemble of magnetic sources M , a magnet being designed, and A , an “anchor” magnet serving as support tool, either virtual or real, for the design and assembly of M . Defining both through their magnetisation M , generating the magnetic field $H_{A}$ and $H_{M}$ in space, the energy reciprocity between the two can be stated as per [Bibr bib1.bibx17]:

1 $$\int H_{M}\cdot M_{A}\text{d}R^{3}=\int H_{A}\cdot M_{M}\text{d}R^{3}.$$

In other words, the magnetic vector potential's equivalent magnetic moment $M_{A}$ has an energy in the magnetic moment's external field $H_{M}$ that is equal to the energy of the original magnetic moment $M_{M}$ in the external field of the vector potential $H_{A}$ . If one assumes high-coercivity magnets with an effective tensorial permeability $\mu_{0}\mu_{r}$ and remanent field $B_{r}$ , i.e. a model which approximates the behaviour of common hard magnetic materials such as NdFeB compounds well, it is possible to set $M=(\mu_{r}-1)H+\mu_{0}^{-1}B_{r}$ . Taking the integration volume $R^{3}$ to volumes where M is non-zero, i.e. $V_{M}$ and $V_{A}$ , one can transform Eq. ([Disp-formula Ch1.E1]) to

2 $$\begin{array}{rl} & \int B_{r_{A}}\cdot H_{M}\;dV_{A}=\int B_{r_{M}}\cdot H_{A}\;dV_{M}\\ & \;\;\;\;+(\int [\mu_{0}(\mu_{r_{M}}-1)\cdot (H_{A}+H_{M})]\cdot H_{A}\;dV_{M}\\ & \;\;\;\;-\int [\mu_{0}(\mu_{r_{A}}-1)\cdot (H_{A}+H_{M})]\cdot H_{M}\;dV_{A}). \end{array}$$

Whereas a solution where only the first two terms hold would yield a formal statement allowing for the superposition of fields, such an interpretation remains an approximation when developing MR applications, as the permeability of samples and magnets will naturally differ. One can nonetheless estimate the validity of this approximation, as it is dominated by the relative amplitude of the term in parenthesis with respect to the first one on the right. Beyond being a differential term with a similar integrand and integration volumes, namely when the magnet and the anchor are similarly sized, both terms are further multiplied by a reductive coefficient, $\left(\mu_{r}-1\right)$ , quite small in the case of neodymium magnets ( $\mu_{∥}=1.03$ and $\mu_{⊥}=1.12$ , [Bibr bib1.bibx18]). This clearly shows how the first term on the right remains dominant, energy-wise, and one can thus approximate the coupling of two magnets through a linear superposition.

**Figure 1 Ch1.F1:**
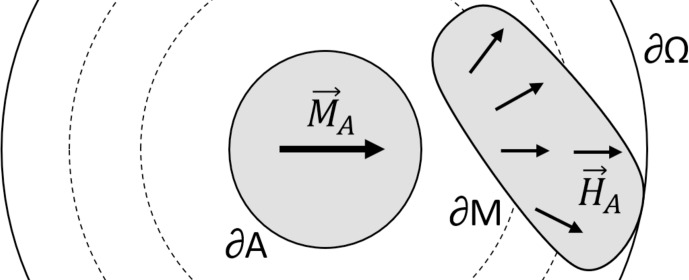
Representation of the domains involved in the reciprocity principle. As an example, the magnet under development, M , outlined by the boundary ∂M of $\Omega_{M}$ , is to create a homogeneous field across the anchor magnet, A , outlined by the boundary ∂A of $\Omega_{A}$ . Reciprocally, the field created by anchor magnet $x_{A}\in \Omega_{A}$ on the magnet volume $x_{M}\in \Omega_{M}$ is shown and defines the direction that would better create a field in A with a uniform direction. In this case, by wanting to create a homogeneous field in a circle, the end solution for the magnet M under development is the well-known Halbach distribution.

Equation ([Disp-formula Ch1.E1]) holds for an arbitrarily small M , which indicates that a higher anchor field in a location will also mean a larger contribution to the target field from magnetisation located there. Similarly, the discussed method does not elucidate the boundary design which leads to an homogeneous target field.

If one defines a target magnetic field to be generated inside the anchor volume as $B_{\mathrm{target}}=-B_{r_{A}}$ , in Eq. ([Disp-formula Ch1.E2]), one can see how maximum compliance with the target field (i.e. $B_{\mathrm{target}}\cdot H_{M}$ being maximal or the field from the magnet under design being parallel to the desired/target direction) corresponds to the minimum magnetostatic energy condition (i.e. $B_{r_{M}}$ and $H_{A}$ being anti-parallel or at the lowest energy orientation). This corollary indicates that a freely rotating magnet will tend to the direction that better generates the target field needed to minimise the energy with the anchor magnet, thus providing the force needed for *self-assembly*.

Just as this effect proves the hypothesis stated in [Bibr bib1.bibx3], used to auto-assemble eight magnets into a Halbach [Bibr bib1.bibx11] array, it also elucidates an important limitation that arises, namely that of the self-interaction of the designed magnetisation distribution. The ideal magnetisation distribution has a minimum energy in the interaction with the anchor magnet but not on its self-interaction energy, which has it evolving to a global energy minimum. For this reason, each individual magnet must be aligned individually, or the self-interaction must be countered with secondary magnetic fields, so as to have a high-fidelity reproduction of the ideal distribution.

### 2.2 Coupling parameter

Knowing the remanent field distribution that maximises the magnetic field in one volume, one can thus compute the efficiency of an array in matching that distribution. In the case shown, the volume that can be filled with magnet material is contained between ∂A and ∂Ω , corresponding to the domain Ω . The outer boundary is defined as the weakest contour line of $H_{A}$ that intersects M , as indicated in Fig. [Fig Ch1.F1], but this definition can be adapted depending on the application and may be replaced by a symmetry line (e.g. profilometry applications can only populate a half-space). On this volume, one can compute the coupling parameter η , which computes how well the magnet under design, defined by its normalised remanent magnetisation $b_{r}=B_{r}/max(|B_{r}|)$ , is aligned with the field created by the anchor, as per

3 $$\eta =\bar{H_{A}\cdot b_{r}}=\frac{\int (H_{A}\cdot b_{r})\;dV_{M}}{\int |H_{A}|\;dV_{\Omega }}.$$

For example, in an ideal 2D Halbach magnet with η=1 , the target field has perfectly circular contour lines, so that Ω is bounded by the least contributing magnetisation, the outer diameter of the magnet array, and the boundary of the circular sample, the inner diameter of the magnet array. These match exactly the placement and direction of magnets in an ideal 2D Halbach array, and the coupling is thus optimal. Real implementations with suboptimal filling factors or supporting structures will however have η<1 . The coupling parameter compares designs quantitatively and provides a computable cost to inefficient but necessary design techniques, like introducing a dead volume to increase homogeneity [Bibr bib1.bibx13], using an anti-aligned piece to cancel first-order field gradients [Bibr bib1.bibx23] or overextending a magnet to emulate an infinite height [Bibr bib1.bibx29].

### 2.3 Arrangement of *n* magnets

A design choice that often directly impacts the performance and complexity of a magnetic arrangement is the number of individual parts involved. A large number of these will self-evidently allow for a better discretisation of a desired magnetisation profile. This introduces the possibility of a stronger magnetic field, by better matching the functional introduced in Eq. ([Disp-formula Ch1.E3]), but the dependence on the experimental effects governing field inhomogeneity remains unclear.

For high-homogeneity applications using permanent magnets, the centre of a 2D discrete Halbach field remains the ideal placement area for a test sample, due to a saddle point of the magnetic field intensity in the axis of the arrangement. A symmetrical placement of four magnets, or a combination thereof, will show a saddle point with zero first and second derivatives of the field intensity. This means that any deviation from an ideally built discrete array can be modelled well, locally, by the total effect of each contribution on its directional, first-order derivative at the origin.

Consider the magnetic field generated by one 2D cylindrical magnet of radius $r_{0}$ and uniform magnetisation $M_{0}$ , as per [Bibr bib1.bibx17], with an angle defined by a rotation matrix $R_{\theta }$ and placed at $\left\{x_{0},y_{0}\right\}$ :

4 $$\begin{array}{rl} B(x,y,x_{0},y_{0},\theta )= & \;M_{0}r_{0}^{2}\mu R_{\theta }\\ & \cdot [\frac{(x-x_{0}{)}^{2}-(y-y_{0}{)}^{2}}{((x-x_{0}{)}^{2}+(y-y_{0}{)}^{2}{)}^{2}}e_{x}\\ & +\frac{2(x-x_{0})(y-y_{0})}{((x-x_{0}{)}^{2}+(y-y_{0}{)}^{2}{)}^{2}}e_{y}]. \end{array}$$

Summing the effect of various cylinders allows for the analytical description of the field generated by an array of n magnets in a Halbach configuration, placed on the unitary circle:

5 $$\begin{array}{rl} B_{H} & \left(x,y\right)\\ & =\;\sum_{i=1}^{n}B(x,y,\mathrm{cos}(2\pi i/n),\mathrm{sin}(2\pi i/n),-4\pi i/n). \end{array}$$

This equation enables the computation of $\nabla |B_{H}|\cdot v$ at the origin, for an increasing number of magnets. Assuming that the experimental uncertainty in magnetisation intensity, angular direction, and piece dimensions has a small relative deviation, $\epsilon_{i}$ , one can use the small angle approximation and obtain the following figures of merit (FOM):

6 $$\begin{array}{rl} {\text{FOM}}_{\Delta \mathrm{Mag}}\propto & {\text{FOM}}_{\Delta r_{0}}\propto \nabla |\sum_{i=1}^{n}(1+\epsilon_{i})B_{i}|\cdot v\\ & =\sum_{i=1}^{n}c_{i}(v)\epsilon_{i} \end{array}$$

7 $$\begin{array}{rl} {\text{FOM}}_{\Delta \theta }\approx & \nabla |\sum_{i=1}^{n}\left(\begin{array}{cc} 1 & -\epsilon_{i}\\ \epsilon_{i} & 1 \end{array}\right)B_{i}|\cdot v\propto \sum_{i=1}^{n}c_{i}(v)\epsilon_{i}+n^{-1}\\ & \cdot \sum_{i,j=1}^{n}k_{ij}(v)\epsilon_{i}\epsilon_{j}. \end{array}$$

In Eqs. ([Disp-formula Ch1.E6]) and ([Disp-formula Ch1.E7]), one arrives at a total inhomogeneity that is dominated by a weighed linear sum of the various effects. Assuming that $\epsilon_{i}$ has a Gaussian distribution around a zero mean, it becomes clear that the field inhomogeneity will increase as $\sqrt{n}$ as the sum of statistically independent distributions of the magnet's behaviour.

Due to a linear superposition, the field scales with the number of magnets, n , while the effects dominating the inhomogeneity of the field, originating from fabrication errors, scale as $\sqrt{n}$ , which means an overall improvement of the relative field inhomogeneity of $1/\sqrt{n}$ . This shows a deep incentive in increasing the total number of magnets in an assembly, which follows common fabrication approaches [Bibr bib1.bibx13].

## 3 Methods

## 4 Result normalisation

Given the scale independence of the laws governing magnetic fields, it becomes natural to normalise all the reported values so that their use is straightforward across materials and application sizes. For this reason, all values shown are presented as adimensional. Magnetic field flux intensity is normalised per unit remanent field $B_{r}$ , meaning field intensity will scale directly with remanence improvements, provided permeability stays constant and coercivity scales accordingly. Dimensions are shown normalised by a constant length, critical to the application, and explicitly defined. The norm of field intensity gradients is normalised by $B_{r}$ and multiplied by the characteristic length, to remove scale/material effects. Magnetic field inhomogeneity was defined as the relative standard deviation of the magnitude of the magnetic field, in a specific volume, and is thus shown in adimensional parts per million (ppm).

### 4.1 FEM solver

The simulations presented were performed in a commercial FEM solver (COMSOL Multiphysics, COMSOL AB) by solving for the scalar magnetic potential, related to the magnetic field intensity as $H=-\nabla V_{m}$ , with $\mu_{0}\nabla \cdot (H+M)=0$ . No placement, magnetisation, or angular errors were considered due to their demonstrably reducible impact. A linear constitutive relation was used, as explained in Sect. [Sec Ch1.S2.SS1], and the validity of the model was checked after each simulation. Built-in routines controlling the convergence of the mesh and numerical error were used to guarantee residual errors below 1 % for all numerical values shown.

### 4.2 Magnetic material model

NdFeB magnets, due to their high coercive strength, are often modelled [Bibr bib1.bibx11] with a constant marginal isotropic permeability ( $\mu_{r}=1.05$ ) and a constant remanent field for $M=(\mu_{r}-1)H+\mu_{0}^{-1}B_{r}$ . This is clear when observing simulated Halbach homogeneity profiles with radially repeating patterns and is a crude approximation of the real non-linear anisotropic response. For this reason, and due to the difficulty in obtaining a complete model for NdFeB magnets, the values used were those reported in [Bibr bib1.bibx18] due to their completeness: $B_{r}=1.15$ T, $Hc_{0{}^{\circ }}=3.0$ T, $Hc_{45{}^{\circ }}=3.0$ T, $Hc_{90{}^{\circ }}=5.6$ T, $\mu_{∥}=1.03$ , and $\mu_{⊥}=1.12$ . These remanence and permeability values along with the directional coercivity were taken as a phenomenological model, shown in Fig. [Fig Ch1.F5]d, and are coherent with the results shown in [Bibr bib1.bibx26]. Experimentally, one can nonetheless employ existing commercial grades of neodymium with better performance characteristics, for improved results.

### 4.3 Energy minimisation

Some of the applications presented require self-alignment of arrays and thus the discovery of the equilibrium position. This is achieved in each case with a magnetostatic energy minimisation subroutine, which finds the local or global equilibrium position. This configuration is then used to compute the behaviour of the field, in each application.

In Sect. [Sec Ch1.S5.SS1], computing the non-corrected discrete Halbach array, a simple routine was used to iterate the angle α . The corrected alignment was then entered directly for comparison, but a check that a correction piece would be possible was performed beforehand.

When extrapolating to a continuous magnetisation distribution, this energy minimisation routine was performed on a scalar field, the angle of the magnetisation distribution on the available volume. The initial values were the Halbach distribution, as only a small perturbation is expected, allowing for a fast convergence. To guarantee this would be the global energy minimum, which is a non-trivial expectation because the magnet becomes much larger than the anchor, symmetry boundary conditions were removed, and the initial condition was set to be the one achieved after uniform magnetisation with a strong external field, as would be the case for a fabrication setting. The algorithm, despite not representing a physical evolution, returned a near-Halbach configuration even for the largest magnet, indicating that it would be the end configuration.

In Sect. [Sec Ch1.S5.SS2], due to the rotational sectional symmetry of the design, only 1 of the 20 sectors was simulated, which assumes simultaneous and identical rotation of all magnets. An energy minimisation routine was used to find the equilibrium rotation angle of the cylinders when specifying the control array at each control position.

### 4.4 Topology optimisation

In Sect. [Sec Ch1.S5.SS3], density-based topology optimisation was employed through the maximisation of the objective function $f_{\text{OBJ}}$ using the solid isotropic material with penalisation (SIMP) method [Bibr bib1.bibx1]. The functional increased the magnetic field norm at height 1, while reducing the non-binary state of the density with the term ρ(1-ρ) , ensuring a sharp transition using the term |∇ρ| and guaranteeing that the magnetic field remains within the constrains of the linear model through the 2D Heaviside function $\Theta (B-\mu_{0}Hc)$ , to avoid demagnetisation:

8 $$f_{\text{OBJ}}=|B|-k_{1}|\nabla \rho |-k_{2}\rho (1-\rho )-k_{3}\Theta (B-\mu_{0}Hc)\rho .$$

The density was linear in the remanent magnetisation and had an initial value of 1. Several steps were used, with various size-dependent $k_{i}$ and mesh density, using both the globally convergent method of moving asymptotes (GCMMA in [Bibr bib1.bibx36]) and sparse non-linear optimiser (SNOPT in [Bibr bib1.bibx10]) algorithms, to allow for convergence of the density.

## 5 Applied reciprocity

### 5.1 Auto-alignment

An impactful application of the reciprocity principle is the automatic assembly targeting micro-scale applications, which, due to the small dimensions and the large relative forces involved, make assembly especially challenging. To best showcase the possible approaches, a micro-array and a powder magnet were simulated in their self-alignment and corrected alignment, attained through application-dependent correction pieces.

A Halbach micro-array was initially conceived to be deposited as a single material layer on a substrate, uniformly magnetised, and allowed to self-align. This enables the use of a low fringe-field design, with a strong field intensity, in integrated applications, while using any desired magnetic material. A quarter of the array was simulated as a 2D model, with sizes ranging up to the maximum packing of eight circles in the available space Ω , between the normalised inner radius and $d_{M}$ .

**Figure 2 Ch1.F2:**
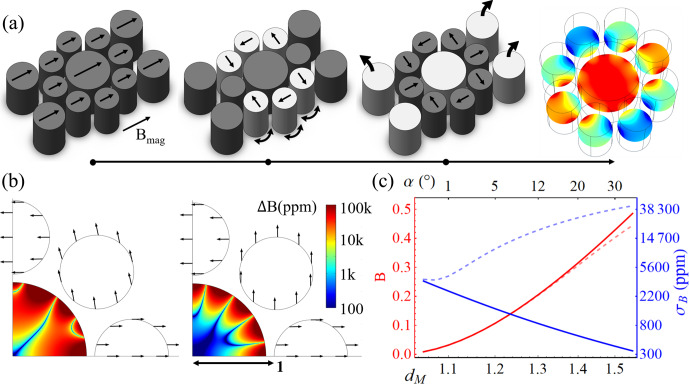
**(a)** The alignment process. Ferromagnetic thin film structures are deposited and magnetised uniformly along the lateral direction. Next, non-aligned structures are released from the substrate and allowed to rotate to their force equilibrium positions, after which they are fixed again to the substrate. Finally, correction structures are released and lifted off, leaving the final field distribution. Structures active in a step are shown brighter, and their magnetisation directions are indicated by straight arrows. Curved arrows indicate movement. The homogeneity of the procedure depicted in **(a)** is shown in panels **(b, c)** for the cases in which outer alignment structures are not **(b)** or are used **(c)**. The line plots in **(c)** reveal the standard deviation of the magnetic field, $\sigma_{B}$ (i.e. the homogeneity), as a function of the outer radius normalised to the design radius, $d_{M}$ , and the tilt angle α , for the self-alignments (dashed lines) and corrected alignments (full lines).

The results in Fig. [Fig Ch1.F2] clearly show the impact of outer radius $d_{M}$ on field strength and homogeneity for one eight-magnet ring, which is further exacerbated as several magnet rings are employed. An improvement of 2 orders of magnitude is seen on the homogeneity, as first-order field gradients cancel out in the correct alignment, thus showing that correction is a critical step. From Fig. [Fig Ch1.F2]a, it is clear that the procedure uses only indirect handling of the magnets and requires only sequential liberation and fixation of magnets. The resulting ease of fabrication and the scale invariance of permanent magnets make these ideal for downscaling magnetic arrays to a length scale far below what is currently achievable for complex arrays.

A natural follow-up to the results shown is the evolution from a finite number of well-defined magnetic structures to arbitrarily many, much like in a powder magnet. These allow for easy tooling, the drastic reduction of statistical variance errors as seen in Sect. [Sec Ch1.S2.SS3] (by shifting these to fabrication precision), and large height micro-magnets, overcoming sputtering height limitations. A powder can reach a maximal theoretical sphere packing density of 74 % in a face-centered cubic (FCC) or hexagonal close-packed (HCP) lattice but, without correct placement, will reach a random packing after vibration annealing, of 64 % [Bibr bib1.bibx16]. A mix of powders of staggered sizes can however reach arbitrarily close to a 100 % packing factor, as smaller powders fill the gaps left by larger pieces or even emulate a single phase with composite effects. Using two powder species with different temperature coefficients of remanence, α , one positive and one negative, it is possible to locally cancel temperature-induced drifts in field strength or even offset volume drifts by tuning the expansion of the binding agent. A thermally compensated mix of NdFeB ( $B_{r}=1.45$ T, α≈-0.12 % K ${}^{-1}$ ) and SmEr ( $B_{r}=0.89$ T, α≈+0.11 % K ${}^{-1}$ in [Bibr bib1.bibx6], for example, shows an effective packing density of 75 % when compared to NdFeB alone.

**Figure 3 Ch1.F3:**
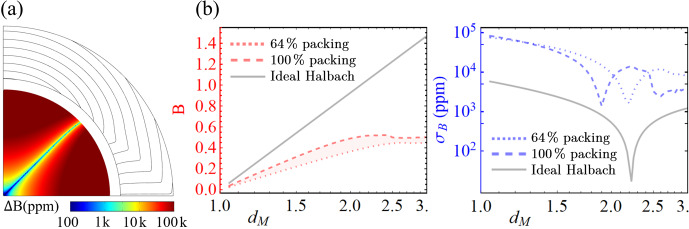
Panel **(a)** shows the implementation of the structures in Fig. [Fig Ch1.F2], in their self-aligned, minimum-energy condition, from powdered material. **(b)** Dependence of field intensity and homogeneity, inside the inner half-radius, on an increasing magnet size, as defined by the outer radius normalised by the anchor's radius, $d_{M}$ .

Using a procedure analogous to the one in Fig. [Fig Ch1.F2]a, Fig. [Fig Ch1.F3] shows that the auto-alignment of a magnetic powder structure is also possible and able to provide a significant field intensity while leveraging all of the advantages mentioned above. Field intensity is seen to level off as the self-interaction of the powder dominates over the alignment field of the anchor, which indicates a need for the use of correction structures. This is further emphasised by the large inhomogeneity gap between the self-aligned and the Halbach conditions. Due to the complexity of the endeavour and its application-specific nature, further investigation of this effect was deemed to be outside of the scope of the present paper. As a baseline, the ideal Halbach alignment was also simulated, which explicitly shows the critical effect of disregarding the marginal susceptibility in hard magnetic materials.

Whereas a full theoretical Halbach possesses zero inhomogeneity, the real response of the magnetic material to the demagnetising field significantly alters this expectation to the behaviour, as shown in Fig. [Fig Ch1.F3]b.

### 5.2 Adjustable operation

One of the advantages of an auto-assembled magnet is the ease at which it can be put together, usually a secondary concern, as homogeneous MR magnets are only assembled once and then used in a static assembly. Similarly, MR profiling magnets are *set* for a specific working condition, and thus field strength, discernible slice thickness, and penetration depth become magnet parameters that cannot be subsequently changed. However, with an auto-adjusting application, one could dynamically tune the operational conditions as needed, making use of the coupling force introduced by a tuning piece.

By solving the inverse problem, the reciprocity principle readily facilitates application development and the interpretation of results. The authors set out to generate an adjustable profiling magnet with a large penetration depth. These magnets, used to analyse the behaviour of a planar 2D slice parallel to the top surface of the magnet, require a field gradient along the penetration depth that is laterally uniform, that is, to have the frequency of the observable spins vary only along the slices but not inside them. As an initial step, relying on reciprocity, an anchor was set at different heights above a plane, below which magnets could be placed. The variation of the field direction on the design region, as the anchor is moved, reciprocally indicates the variation the magnets need to have to adjust to a target field at different penetration depths, as illustrated in Fig. [Fig Ch1.F4]a, with rotating cylinders. This rotation can then be induced mechanically or with a magnetic control piece. Once more leveraging the principle, the contribution of each point in space to the field on the anchor space and on the cylinder space can be computed. With these, one can optimally find the region in space which strongly interacts with the cylindrical array, inducing torque, but it has a minimum contribution to the target field, reducing any adverse effects. These pieces were implemented as cubes, for simplicity, as seen in Fig. [Fig Ch1.F4]a.

The application requires a sectional symmetry to generate the nearly axially symmetric field which allows for a minimal discernible slice. As the alignment of all magnets at the same rotation angle is a meta-stable configuration of the cylinder array, a scaffold is needed to maintain the relative angular positions. A stable configuration of the coherent cylinder array is a parallel orientation pointing upwards/downwards, and, due to the desired high packing density, the strong interaction between the cylinder array elements makes it hard to shift the configuration away from its minimum energy alignment. For this reason, a set of control pieces with identical upward magnetisation is placed between the polarising magnets, creating a local energy minimum somewhat shallower than the absolute minimum, which allows for adjusting the strongly aligned magnet array with smaller pieces. The figures of merit of the profiling magnet assembly are its field strength, its penetration depth, and the discernible slice thickness. The minimum discernible thickness comes from the condition that the frequency variation along the penetration depth be greater than that within a slice of thickness T (i.e. $T\gamma \nabla_{z}B>\Delta \omega_{\text{slice}}$ ) and was implemented, for the field in a cylindrical slice of radius R , as

9 $$T_{\text{min}}(R,z)=\frac{\text{Max}(\Delta |B|){|}_{r<R}}{\text{Min}(\nabla_{z}|B|){|}_{r<R}}.$$

Due to the scale invariance of the field profile, all dimensions were normalised to the outer diameter of the available design volume (i.e. the magnet), and the geometry was obtained as follows.

From the results in Fig. [Fig Ch1.F4] and Table [Table Ch1.T1], the achieved results, beyond their novel continuous-tuning ability, outperform other reported designs and simulations in penetration depth, while having similar values of discernible thickness and field intensity/gradient, when compensating for the unavoidable distance decay. Further optimisation on the packing density was not attempted, as limits are fabrication-dependent, and the centre was left unpopulated to allow for radio-frequency (RF) coils, meaning the performance could be increased further significantly.

**Figure 4 Ch1.F4:**
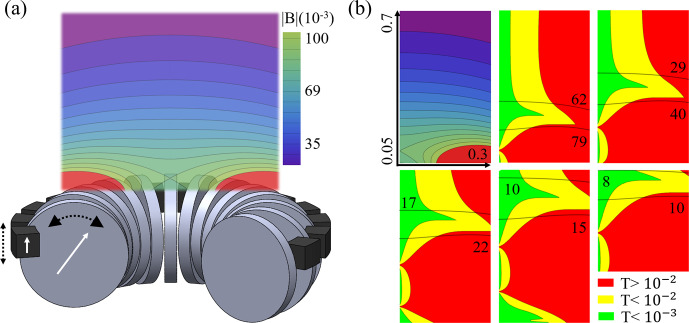
**(a)** Adaptable magnet geometry, with a section removed for clarity, shows a toroidal array of disk magnets in grey, magnet control pieces in black, and the magnetisation direction as white arrows. The resulting magnetic field intensity contours are shown after having reached the minimum energy configuration and feature a region above the magnet array with a laterally uniform gradient along the depth. In **(b)**, the top-left and top-centre plots show the field contours and minimum discernible thickness for the magnet configuration in **(a)** as defined by Eq. ([Disp-formula Ch1.E9]). The remaining minimum discernible thickness profiles show other equilibrium configurations arising from moving the control pieces in z∈{-0.06,0.08} , thereby rotating the cylinders by $\theta \in \{-32{}^{\circ },27{}^{\circ }\}$ . These are plotted on normalised dimensions going from 0.05 to 0.7 in relative penetration depth and 0 to 0.3 radially. The normalised magnetic field intensity, in normalised milliunits, and its contour lines, are shown in black.

**Table 1 Ch1.T1:** Normalised figures of merit, as described in Sect. [Sec Ch1.S3], comparing the obtained results for the normalised penetration depth ( d ), magnetic field ( B ), normalised field gradient, and minimum discernible thickness ( T ) to literature.

	d (%)	B	$\nabla_{z}|B|$	T ( $10^{-3}$ )
Figure [Fig Ch1.F4]	25–68	0.008–0.07	0.2–0.3	<1
[Bibr bib1.bibx31]	25	0.18	0.36	0.7
[Bibr bib1.bibx21]	19	0.36	0.26	0.3
[Bibr bib1.bibx4]	3	0.22	0.94	0.3
[Bibr bib1.bibx24]	7	0.07	0.02	1.8

### 5.3 Optimised coupling

Beyond the applications shown, which emphasise fabrication and readjustment, the principle shown here is especially well suited for the design of strong magnets, as it near-optimally removes the degrees of freedom associated with angular alignment and mass-optimal magnet placement, which allows for development through shape/topology optimisation.

As the field of spectroscopic NMR evolves, with experiments now being done in volumes below $(5\;\mathrm{nm}{)}^{3}$ using NV centre spectroscopy [Bibr bib1.bibx35], superconducting magnets remain the most expensive and obtrusive element of the experimental setup. As an alternative, we set out to create the strongest possible field using permanent magnets in a geometry that allows for easy access, such as a magnet integrated into a laboratory table, and with axial symmetry, to allow for easier fabrication and assembly. Such a setup would enable a passive, low-cost magnet with an encapsulation allowing for temperature control and multidirectional access to optical instrumentation or sample feed lines.

A critical limitation of external fields is that of strong gradients, as the field decays away from the magnet. Approaches based on creating a saddle point were researched and, when compared to the achieved solutions, were found to have a drastically reduced magnetic field intensity. Given the targeted application of small-scale experiments, the use of electromagnetic coils to correct for the axial gradient is far better suited, considering the scaling of field gradients and power consumption at reduced scales.

As a starting point, magnetostatic reciprocity presets the optimal magnetisation direction on the half-space, a dipole field [Bibr bib1.bibx33] centred at a normalised height of 1 above the plane and defined by its polar angle θ :

10 $$\begin{array}{rl} B_{d}= & \;\frac{\mu_{0}}{4\pi }\frac{\left|m_{z}\right|}{(\rho^{2}+(z-1{)}^{2}{)}^{3/2}}\\ & \cdot [1.5\mathrm{sin}(2\theta )e_{\rho }+(3{\mathrm{cos}}^{2}(\theta )-1)e_{z}]. \end{array}$$

The contour lines for the intensity of the field determined the optimal placement of the magnets to be a half-ellipsoid with a smallest axis/radius of $R_{M}$ . This result is reminiscent of a Halbach configuration, which is known to have a logarithmic dependence on the outer radius. However, these must maintain a fully packed geometry to achieve homogeneity and thus quickly generate a demagnetised area with larger outer radii [Bibr bib1.bibx14], which limits field intensity. To overcome this, a routine was established to optimally remove voxels which would become demagnetised. As the removal of a volume sharply impacts the demagnetising field on the nearest neighbours, an iterative algorithm was necessary, and thus density-based topology optimisation techniques were used, obtaining the results shown in Fig. [Fig Ch1.F5].

**Figure 5 Ch1.F5:**
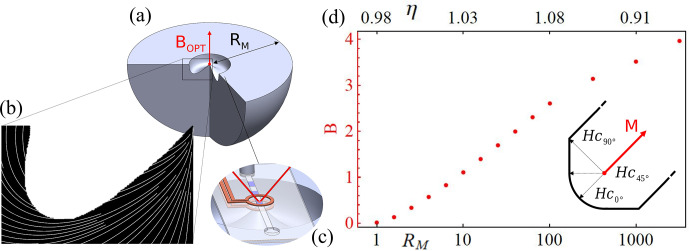
Panel **(a)** shows a section of the geometry (not drawn to scale) of a table magnet that creates an optimal field at the tip of its on-axis protrusion, indicated by a red dot. **(b)** Result of the topology optimisation for a magnet with diameter $R_{M}=10^{3}$ , with the magnetisation direction shown as white streamlines. **(c)** An example application for microfluidic NMR. **(d)** The dependence of the field intensity and coupling parameter η defined in Eq. ([Disp-formula Ch1.E3]), on the normalised (with respect to the distance above the plane being optimised) magnet radius $R_{M}$ . The inset on the right shows the phenomenological demagnetisation model for the magnetisation M used during optimisation. Further details on the model and its implementation as a regularisation are given in Sect. [Sec Ch1.S3], in the magnetic material model and topology optimisation subsections.

The field intensity is seen to quickly attain a Halbach-like logarithmic scaling as the magnet becomes large ( $R_{M}>2$ ) compared to the normalised height and maintains the logarithmic growth, albeit less pronounced, as demagnetised regions start being removed ( $R_{M}>100$ ). The coupling parameter explained through Eq. ([Disp-formula Ch1.E3]) is especially well suited to quantitatively evaluate designs focused on intense magnetic fields. Before removal of magnetic material, the results are close to unity and subsequently diminish as volumes with a large effect must be removed to avoid demagnetisation, showing the reduced efficiency of further increasing the amount of magnetic material. Values of η above 1, while generally unexpected, are caused by the demagnetising field direction matching the alignment of the magnetisation at the centre/tip of the design, constructively magnetising that region. These results open a new avenue towards low-cost, high-performance spectroscopy and can easily be generalised, due to their scale independence, to any field requiring strong localised magnetic fields, such as those needed for magnetic resonance force microscopy.

## 6 Conclusions

The results presented in this paper offer several insights into the development of permanent magnet systems for MR. By establishing a quantifiable metric for what constitutes a good magnet design, a beneficial dependence on the use of multiple magnets, and a way to auto-align them, along with some exemplary applications of these methods, the authors hope to facilitate the future development of high-performance designs and to promote the integration of advanced numerical and experimental techniques into a now further constrained problem.

As discussed, the reciprocity principle establishes a method for developing an intensity-optimal magnet, which should be thought of as the ultimate goal of the MR magnet designer, as the field's homogeneity can then be targeted through a wealth of solutions. Despite its broad applicability and usefulness, the original principle harboured an intrinsic limitation by relying on the superposition of linear responses, which breaks down when strong non-linear effects are present. Unfortunately, these are central to state-of-the-art solutions, which nevertheless have generated impressive results up to 4 T [Bibr bib1.bibx20], by leveraging the saturation magnetisation of soft-magnetic materials of up to 2.8 T [Bibr bib1.bibx27]. On the other hand, the breakdown of linearity poses severe challenges in the development of shimming systems, and design trade-offs must thus be made.

The results further emphasise the need for a large filling factor of the magnet, as this has a large effect on the magnet's coupling parameter and thus volume and cost. This approach requires a removal of in-bore passive shimming systems, which must be placed outside, or the use of other approaches. External shimming systems could benefit from the negligible fringe field of Halbach magnets, for example, or even aid with the reduction of the demagnetisation field. Alternatively, given the extreme homogeneity values required for spectroscopy, other approaches are likely to dominate given the lack of fabrication procedures at high enough precision and the ability to target constant field deviations through other means. As sample sizes become smaller, the use of active shimming becomes more favourable as the power efficiency of shimming coils increases and the absolute power dissipation decreases, with an approximate dependence of $l^{-1}$ [Bibr bib1.bibx19]. If the magnet size remains constant and the sample/shims are downscaled, a linear shimming profile generating the same field will span a larger gradient in the smaller enclosed volume, which introduces a further geometrical scaling factor of $l^{-1}$ .

On the other hand, field inhomogeneity does not constitute a fundamental problem for NMR, as it only reduces the net measured signal and not the local contributions. This effect comes naturally from local dephasing, which has successfully been targeted with “shimming” RF pulses [Bibr bib1.bibx38], which can periodically or continually compensate for any local deviation in phase.

Lastly, the authors targeted self-aligning arrays, which have long been an interesting phenomenon that can now be brought to MR by leveraging two of their intrinsic advantages. While self-interaction will always pose a constraint, requiring correction, a large part of the magnetostatic energy and thus the forces involved are already associated with the desired effect, through the use of an anchor magnet. This means that the forces associated with correction and assembly, even when manual, can be significantly reduced, making these two-step processes easier, as shown by the simple correction pieces employed in Fig. [Fig Ch1.F2], which easily solve the limitations mentioned in [Bibr bib1.bibx3]. Additionally, the ability to auto-align an array appears to be the only way to break free from deviations caused by the use of a limited number of magnets, which in turn limit the overall repeatability of designs, leaving them susceptible only to assembly/scaffold-fabrication offsets.

## Supplement

10.5194/mr-2-607-2021-supplement The supplement related to this article is available online at: https://doi.org/10.5194/mr-2-607-2021-supplement.

## Data Availability

No raw data were used apart from the cited material parameters introduced in the COMSOL simulations.

## Code availability

COMSOL files used to obtain all results shown are attached in the Supplement.
