# Supplementary material for: Magnetostatic reciprocity for MR magnet design
Source: Magn Reson (Gott). 2021 Aug 4;2(2):607–17. doi: 10.5194/mr-2-607-2021 (PMC10539805; doi:10.5194/mr-2-607-2021)
Supplement: The supplement related to this article is available online at: https://doi.org/10.5194/mr-2-607-2021-supplement. [file mr-2-607-supplement.zip › mr-2-607-2021-supplement-title-page.pdf]

Supplement of Magn. Reson., 2, 607–617, 2021
https://doi.org/10.5194/mr-2-607-2021-supplement
© Author(s) 2021. CC BY 4.0 License.

Supplement of

Magnetostatic reciprocity for MR magnet design

Pedro Freire Silva et al.

Correspondence to: Jan G. Korvink (jan.korvink@kit.edu)

- mr-2-607-2021-supplement-title-page.pdf
- 1st experiment - discretehalbach.mph
- 2nd experiment - powder magnet energy min - Kopie.mph
- 3rd experiment - adjustable profiling cylinders.mph
- 4th experiment - NV magnet.mph

The copyright of individual parts of the supplement might differ from the article licence.
